# Professional development in evidence-based practice: course survey results to inform administrative decision making

**DOI:** 10.5195/jmla.2019.628

**Published:** 2019-07-01

**Authors:** Deborah L. Lauseng, Carmen Howard, Emily M. Johnson

**Affiliations:** Assistant Professor and Regional Head Librarian, Library of the Health Sciences-Peoria, University of Illinois at Chicago, Peoria, IL, dlauseng@uic.edu; Instructor and Regional Health Sciences Librarian, Library of the Health Sciences-Peoria, University of Illinois at Chicago, Peoria, IL, choward4@uic.edu; Assistant Professor and Regional Health Sciences Librarian, Library of the Health Sciences-Peoria, University of Illinois at Chicago, Peoria, IL, emj11@uic.edu

## Abstract

**Objective:**

To understand librarians’ evidence-based practice (EBP) professional development needs and assist library administrators with professional development decisions in their own institutions, the study team surveyed past participants of an EBP online course. This study aimed to (1) understand what course content participants found valuable, (2) discover how participants applied their course learning to their work, and (3) identify which aspects of EBP would be beneficial for future continuing education.

**Methods:**

The study team distributed an eighteen-question survey to past participants of the course (2011–2017). The survey covered nontraditional demographic information, course evaluations, course content applications to participants’ work, additional EBP training, and EBP topics for future CE opportunities. The study team analyzed the results using descriptive statistics.

**Results:**

Twenty-nine percent of course participants, representing different library environments, responded to the survey. Eighty-five percent of respondents indicated that they had prior EBP training. The most valuable topics were searching the literature (62%) and developing a problem, intervention, comparison, outcome (PICO) question (59%). Critical appraisal was highly rated for further professional development. Fifty-three percent indicated change in their work efforts after participating in the course. Ninety-seven percent noted interest in further EBP continuing education.

**Conclusions:**

Survey respondents found value in both familiar and unfamiliar EBP topics, which supported the idea of using professional development for learning new concepts and reinforcing existing knowledge and skills. When given the opportunity to engage in these activities, librarians can experience new or expanded EBP work roles and responsibilities. Additionally, the results provide library administrators insights into the benefit of EBP professional development.

## INTRODUCTION

Health sciences librarians are actively engaged in evidence-based practice (EBP) instruction across several disciplines and health care settings. New openings for integration and engagement are often recognized, yet the availability of training to facilitate meeting these potentials varies widely. The development of EBP knowledge and skills is accessible through a number of professional development avenues: learning on one’s own, learning on the job, learning from colleagues, or learning through formal workshops and courses. Unfortunately, not all EBP training opportunities are equally available for individual librarians, particularly formal professional continuing education (CE) courses.

From an administrative perspective, ensuring that librarians who are involved with EBP instruction and practice have the appropriate skills and knowledge to effectively meet the demands of their work is imperative and should not be overlooked [1]. Recent literature emphasizes the value of facilitating professional development among academic librarians and the benefits of enhanced knowledge about health care processes for EBP instruction [2–4]. Administrative support of professional development can include facilitating internal opportunities and investing in external training workshops and courses to meet a specific EBP need or to introduce an innovative initiative. Professional development is also important for librarian career advancement and for organizational movement forward [5].

Likewise, librarians recognize the critical need for EBP knowledge and the challenge in obtaining these skills to support and expand their engagement with EBP [1, 4, 6]. They understand the benefit of professional development and seek opportunities for training that range from self-directed learning activities to formal CE courses [2, 4]. Application of new EBP knowledge and skills gained through professional development leads librarians to expand their roles in EBP activities [7], be more fully engaged in EBP practice [1], and expand EBP instructional methods and empower learners [3, 8]. Librarians who participate in professional development are also able to increase their productivity, impact their career advancement, and affect their job satisfaction [9]. However, gaps still exist in the support for and availability of EBP professional development, even though administrators and librarians understand the importance and long-term impact of EBP training.

Supporting the development of EBP skills in the profession, the University of Illinois at Chicago (UIC) Library of the Health Sciences (LHS) developed a successful multiday in-person training course that has been taught for more than eleven years [7, 10]. In 2010, the library instructional team applied for and received funding from the National Network of Libraries of Medicine Greater Midwest Region to convert the in-person course to an asynchronous, self-paced, online course offered through the Medical Library Association (MLA) for broader reach to health and information professionals [10]. Through collaborative efforts of the instructional team, including both librarians and physicians, the re-envisioned EBP online course, hereafter referred to as the course, was piloted in the spring of 2011.

The course was offered once per year—with the exception of the first year, which included a pilot—and each cohort was limited to twenty-five participants. The most recent course content included the units:

- “Evolution of EBP”
- “Research Study Methodology”
- “Developing the Answerable Question Using Problem, Intervention, Comparison, Outcome (PICO)”
- “Searching for the Evidence in the Literature”
- “Introduction to Critical Appraisal”
- “Use of Critically Appraised Topics (CATS)”
- “Critically Appraising the Diagnostic Literature”
- “Critically Appraising the Therapy Literature”

Six cohorts of professionals participated and completed the online course as of the last offering in winter 2016/17.

The online instructional videos, readings, and assignments continued to be revised and updated as the instructional team members varied with staffing changes over the six-year period. Most updates were minor content changes and adjustments to assignments. The largest change was an expansion of the “Research Study Methodology” content.

The study team designed and conducted a survey during the spring/summer of 2017 to assess the value of the course and the impact of the learning that had occurred. The research investigation had three aims: (1) to learn what course content participants found valuable, (2) to discover how participants applied their course learning to their work, and (3) to identify additional aspects of EBP that would be beneficial for future CE. Analysis of the collected data would also inform the LHS administrators’ decisions concerning new directions for the course. Additionally, insights gained from this research into the value of specific EBP topics can assist other library administrators with professional development planning in their own institutions.

## METHODS

Eligible research participants completed the course between the years of 2011 and 2017. The study team created a contact list of 122 eligible participants from previous students of the course with updated email contact information that was collected from publicly available contact sources.

The survey collected nontraditional demographic data including work environment, degrees earned, and past EBP training. To understand the impact of the course, participants evaluated the course components and how they applied the course content to their professional work. For gauging future interest in CE, the survey asked if they were interested in any additional EBP topics for future CE. The UIC Institutional Review Board deemed the project exempt from review.

The survey tool selected was Qualtrics [11]. The survey contained eighteen questions using multiple-choice, multiple-answer, or free-response formats (supplemental appendix). The survey integrated the informed consent statement, in which participants were able to indicate their willingness or unwillingness to participate in the study. If participants consented, they progressed to the research survey questions.

The study team entered a panel of eligible participants into the Qualtrics contacts mailing list and prepared a customized invitation letter for distribution. The survey was distributed via email in Qualtrics to participants at least 6 months after they completed the course. The distribution occurred in 2 rounds: first to 100 participants of the spring 2011 through the spring 2015 cohorts and then to 22 participants of the winter 2016/17 cohort. For both rounds, the survey was available for 30 days. Three reminders were sent: 12 days out, 25 days out, and the day before the survey closed.

The study team analyzed the survey results using descriptive statistics. Data were processed using Microsoft Excel [12] and Qualtrics.

## RESULTS

Current contact information was available for 122 past participants of the course. Surveys were distributed to 119 participants, as 3 surveys were returned as “undeliverable.” Thirty-nine participants started the survey. However, only 34 surveys were determined to be usable, with 2 participants never submitting their responses and 3 participants only answering the demographic questions. The final response rate was 29%.

Respondents were distributed across the 6 years of course cohorts. The largest response group was the winter 2016/17 cohort, with 8 respondents. The smallest response groups were spring 2011 and spring 2012, with 3 respondents each. All respondents had advanced degrees. Thirty-two respondents (94%) had a master’s in library science, 1 (3%) had a master’s in nursing, and 1 (3%) had a medical doctor (MD) degree.

**Figure 1:**
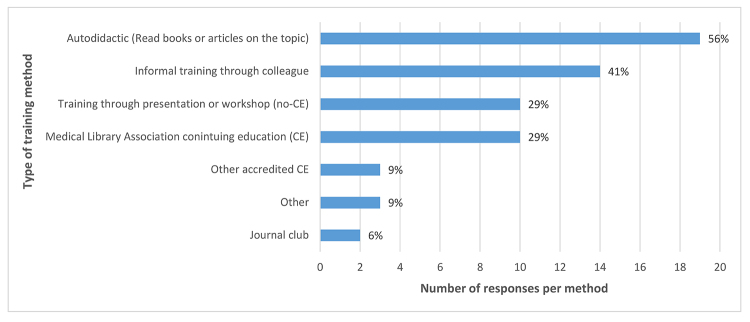
Prior evidence-based practice (EBP) training methods

At the time of the course, respondents were working in academic health sciences libraries (n=13, 38%), hospital libraries (n=13, 38%), academic non–health sciences libraries (n=6, 18%), and other work environments (n=2, 6%). Other work environments were self-reported as “professional association” and “federal, health research.” Survey respondents were asked to indicate all types of EBP training methods that they had participated in prior to taking the course. Twenty-nine (85%) respondents indicated at least 1 form of EBP training. However, only 16 (8%) received EBP training as part of their degree programs. Over half of respondents read books and articles on the topic. The next most popular methods were informal training from colleagues and attendance at formal training, which included CE credits from MLA or another professional organization. Almost a third of respondents had attended formal training that did not include CE credit. A small percentage had been involved in journal clubs or other training opportunities (Figure 1).

**Figure 2:**
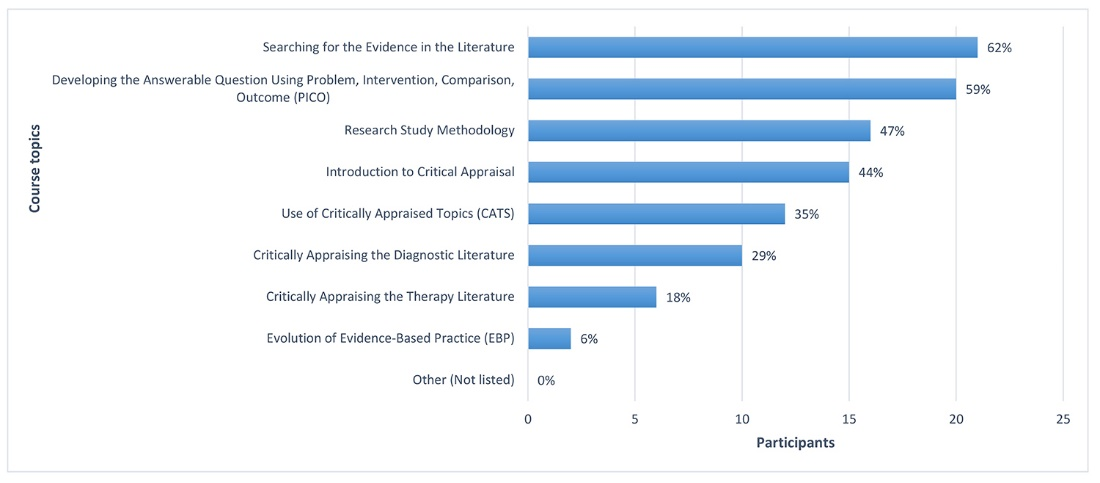
Most valuable topics

When asked to select the top three most valuable topics in the course, over half of respondents selected “Searching for the Evidence in the Literature” and “Developing the Answerable Question Using PICO.” “Research Study Methodology” was selected by nearly half of the respondents. Several respondents selected various aspects of critical appraisal; for example, nearly half selected “Introduction to Critical Appraisal.” However, the “Evolution of EBP” topic was selected by only a couple of respondents (Figure 2).

Similar results were obtained when respondents were asked to select all course topics they had applied to their work since taking the course. “Searching for the Evidence in the Literature” was most frequently selected (76%, n=26), and “Evolution of EBP” was least frequently selected (15%, n=5). The only exception to this similarity was “Use of CATS,” also selected by only 5 (15%) respondents, placing it in a tie for least often applied to work. These results are supported by open-ended responses that indicated primary takeaways for the course as: “Better search skills and MUCH better understanding of how to appraise studies,” “Really defining and refining the PICO,” and “Learning about the different types of studies and how to critically evaluate them.”

Respondents were asked 4 questions related to changes in their work efforts after participating in the EBP course. Respondents could select “yes” or “no” regarding new responsibilities, new work roles, new jobs, or other work changes (Table 1) and had an opportunity to leave an open-response describing any changes. Out of the 34 respondents, 18 (53%) selected “yes” to at least 1 of the questions related to work change. Several respondents indicated new work responsibilities, such as conducting literature reviews and being involved in EBP instruction. Specific examples include:

[I] was able to apply [the] topic of Critically Appraising the Diagnostic Literature to a guideline project.

[I] added a section on the evolution of EBP to the Nursing online orientation.

I used Research Study Methodology in several of my classes to discuss types of studies and with people looking to perform systematic reviews.

I support the EBM curriculum [in] our Department of Medicine where I meet with 3–4 residents 3 times a year where I utilize many content [topics] from the class.

**Table 1:** Indicated work changes

Work change	Yes		No		Blank response	
New work responsibilities	9	(26%)	21	(62%)	4	(12%)
New job	3	(9%)	25	(74%)	6	(18%)
New work role	2	(6%)	24	(71%)	8	(24%)
Other work change	7	(21%)	17	(50%)	10	(29%)

A few respondents specified taking a new position, with one expressing how “familiarity with EBP allowed me to seek out a new position.” A couple indicated a new role in their organizations, including a promotion from a “Library Assistant to a Health Sciences Librarian.” Some respondents also selected other changes in work, noting “better understanding of [evidence-based medicine] EBM,” “greater confidence and improved services for patrons,” seeking self-development of skills, and an authorship opportunity on a systematic review. Overall, the survey respondents provided statements indicating the advantage of participating in the course through examples of how they applied the course content to their work.

**Figure 3:**
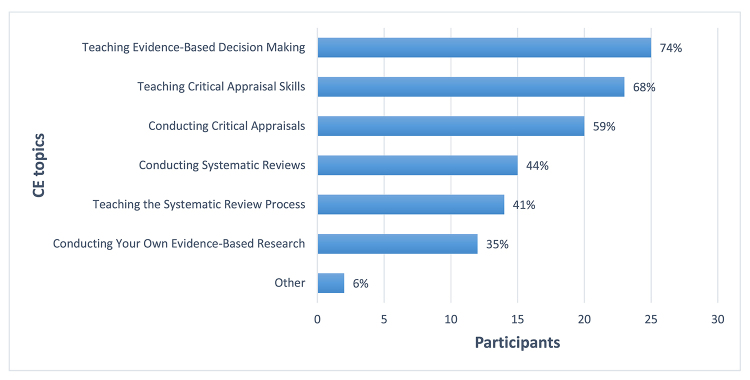
Topics of interest for future continuing education (CE) opportunities

Respondents were asked about their interest in six potential topics for future CE opportunities. Topics reflected a focus on either teaching EBP to others or actually performing EBP tasks. The highest level of interest was in the topic of “Teaching Evidence-Based Decision Making,” closely followed by “Teaching Critical Appraisal Skills” (Figure 3). The other popular topic was “Conducting Critical Appraisals.” The remaining topics, selected by less than half of the respondents, were “Teaching the Systematic Review Process,” “Conducting Systematic Reviews,” or “Conducting Your Own Evidence-Based Research.” The higher interest in the two teaching topics was supported by the open responses that indicated a primary goal of teaching for many respondents. Respondent comments included:

I would like to be able to add it to my 2nd year med students’ EBM lecture, but I really don’t feel like I have [a] good handle on it.

[H]elped with better understanding of EBM and enabled me to be a better instructor and facilitator of EBM.

Additionally, when given the opportunity to suggest their own topics, respondents replied with the following ideas “Patient perspectives of EBP,” “Critical appraisal of EBP for consumer health,” “incorporating narrative medicine in evidence-based practice,” and “advanced systematic reviews.”

## DISCUSSION

Many examples exist of librarian engagement with EBP instruction in the health sciences disciplines [13–17]. Previous studies [1, 4, 6] indicate that librarians seek development and enhancement of their EBP knowledge and skills, particularly those involved with EBP instruction. Librarians recognize the value of expanding their expertise, equipping themselves to address or anticipate the demands and opportunities that are available for their integration with EBP educational activities. Our survey results are consistent with previous research showing that respondents desire further development of teaching EBP skills.

With broad integration of EBP into health sciences librarian roles, the study team expected that basic EBP concepts like working with a PICO question and advanced searching skills would be competencies that librarians already held and that respondents would not highly value. The high value placed on certain course components was unexpected, specifically “Developing the Answerable Question Using PICO” and “Searching for the Evidence in the Literature.”

Respondents’ comments revealed that the course content helped those who were new to EBP develop “a basic understanding.” Other comments indicated that the course helped with “reinforcing what I had previously learned” and “helped with a better understanding [of EBP].” These comments, and the fact that 85% of respondents had previous EBP training, strengthened the case for librarians participating in both foundational and advanced EBP educational opportunities. Further highlighting this idea were responses that specifically stated the need to learn more, such as “that as much as I learn about EBP, there is always more that I need to learn,” and the need to keep up to date, such as “[the course] reinforced the need to review certain content as well.” Respondents also expressed that online courses with hands-on exercises “should be offered more often to keep up with these skills.”

As anticipated, the critical appraisal components of the course were also highly valued; however, respondents’ comments suggested that critical appraisal topics were not fully understood. The findings showed that a higher number of respondents valued the “Introduction to Critical Appraisal” topic more than any one of the specialized appraisal topics included in the course, for example, “Critically Appraising the Therapy Literature” or “Critically Appraising the Diagnostic Literature.” This result might support the ongoing need for more knowledge in this aspect of EBP for librarians. The application of critical appraisal varied among survey respondents. Some respondents noted feeling comfortable integrating teaching appraisal into their work, whereas several felt they needed further training to build their confidence in incorporating this aspect of EBP into their instructional activities. Additionally, critical appraisal was highly rated as an area for further professional development, reinforcing the idea that respondents valued this content but felt that they had a limited understanding of the topic.

Librarian participation in professional development is readily acknowledged as vital for growth of professional competence and career advancement [5, 9]. The benefits of professional development have long been valued by administrators, particularly the application of new learning in the workplace. The retention and application of new EBP skills is important for evolving EBP roles and enhancing EBP practice [1, 4, 7].

As detailed in the results, several respondents provided statements about how they applied their course learning to their work. One respondent summed up their application of learning with this statement:

I have been able to more confidently teach live classes on these topics thanks to the formal training in this class. With the Critical Appraisal, in the past I knew about it but had never done one myself. Now, having done one I can appreciate the parts where students are struggling and recognize the points of struggle a bit more. This has made me a more effective teacher.

The fact that more than half of respondents indicated some level of work change (e.g., new responsibilities, roles, or job) combined with the qualitative open-ended responses reinforces the value that librarians place on participating in EBP professional development. The anecdotal evidence also provides administrators with specific examples of successful application of new EBP skills resulting from participating in a professional development course.

Library administrators and librarians alike need to recognize the significance of developing EBP expertise over time through continuing professional education. EBP training is important for establishing foundational understanding as well as for expanding the level of EBP engagement. Further insight into librarians’ interest in continuing their EBP education can be drawn from the survey responses about training prior to the course and interest in additional training following the course. Most respondents indicated that they participated in some form of EBP training prior to the course, and nearly all noted that they were interested in additional EBP CE after participating in the course. Respondents selected two aspects of teaching EBP at a higher frequency than any aspect of conducting EBP practice from the list of potential EBP CE topics. The lower frequency of selecting the CE option for practicing EBP might reflect the need for additional understanding of incorporating EBP into the disciplinary practice of librarianship [18].

Professional development can occur through several methods, including learning on one’s own, learning on the job, learning from colleagues, or learning through formal workshops and courses. The delivery mechanisms for formal training, whether for CE or not, include in-person workshops, self-paced tutorials, online courses, or open educational resources. In regard to past EBP training, respondents indicated that most of their informal learning was self-directed through reading articles and books or interacting with library colleagues. Respondents indicated that formal learning occurred through non-CE or CE workshops and classes. While respondents’ reasons for participating in a particular learning method were unknown, recent studies have suggested that librarians preferred “the freedom to choose their professional development activities” [2] and the “ability to self-direct their learning” [4].

Insights gained from the survey responses are informative for the course administrators who are considering alternate methods for delivering updated and expanded EBP professional development. For other library administrators, understanding the varied EBP training methods that are available can facilitate their approaches to assuring professional development at the level necessary for librarians to meet their users’ EBP demands. Administrators look for professional development opportunities for many reasons, such as allowing growth in positions and roles and developing expertise to enhance services and integration.

In addition to the support of library administrators, the support of professional organizations is also vital. For example, MLA has placed a growing emphasis on EBP. In the 2007 *MLA Professional Competencies for Health Sciences Librarians*, EBP concepts were integrated throughout the seven competencies [19]. The *2017 MLA Competencies for Lifelong Learning and Professional Success* have a specific EBP and research competency [20]. Additionally, MLA has revitalized its education agenda for its membership, having recognized the essential need for ongoing professional development, particularly to support the stated professional competencies.

### Further research

Further research, beyond anecdotal evidence, on the application of new skills and associated work changes resulting from participating in formal professional development would be valuable to library administrators and librarians alike. Related research into librarian preferences for professional development instructional methods would inform those who create formal CE sessions as well as those who develop in-house professional development programs. With the interest in additional EBP training, a framework for the quantity and scope of formal EBP training is needed for librarians as they continue to develop and expand their engagement in EBP. Investigating whether librarians are more interested in teaching aspects of EBP versus conducting their own evidence-based librarianship practice would also inform this framework.

### Limitations

There were limitations to this study. The survey had a small sample size of participants and no control group, limiting generalizability of results. The survey design and question format were based on nominal scales, which only allowed descriptive analysis and reporting. Participants were asked to reflect on course content and past experiences for events up to six years prior to the survey, introducing potential recall bias or error. The participants also self-reported their own behavior and actions, so the behaviors they reported might not be representative of participants’ real-life activities.

## CONCLUSION

Survey respondents found value in both familiar and unfamiliar EBP topics. The value assigned to these topics supported the idea that professional development should be used not only to learn new concepts like critical appraisal, but also to reinforce existing knowledge and skills such as developing PICO questions and searching the literature. When given the opportunity to engage in professional development activities, librarians can experience new or expanded work roles and responsibilities. Specific examples of this application to work varied from simply expanding the topics taught in EBP instructional sessions to seeking out and securing a new position. Respondents showed a continuing interest in CE opportunities even when they had participated in multiple learning activities. This interest was predominately for additional training in the teaching aspects of EBP rather than in personal practice. Our survey results provide library administrators insights into the need for continuing EBP professional development in order to establish foundational knowledge and to expand skills for increasing librarian EBP competence and improving EBP services.

## SUPPLEMENTAL FILE

Appendix Past evidence-based practice (EBP) course participants survey, 2017
