# Supplementary material for: Professional development in evidence-based practice: course survey results to inform administrative decision making
Source: J Med Libr Assoc. 2019 Jul 1;107(3):394–402. doi: 10.5195/jmla.2019.628 (PMC6579596; doi:10.5195/jmla.2019.628)
Supplement: Appendix [file jmla-107-394-s001.pdf]

JMLA Journal of the Medical Library Association

SC

Supplemental content to
J Med Libr Assoc. Jul;107(3):dx.doi.org/10.5195/jmla.2019.628
www.jmla.mlanet.org
© Lauseng, Howard, Johnson 2019

Professional development in evidence-based practice: course survey results to inform administrative decision making

Deborah L. Lauseng; Carmen Howard; Emily M. Johnson, AHIP

APPENDIX

Past evidence-based practice (EBP) course participants survey, 2017

What environment were you employed in when you attended the evidence-based practice (EBP) course? (select one)

- O Public library
- O Academic library (other than health sciences)
- O Academic health sciences library
- O Hospital library
- O Nonlibrary clinical/health care setting
- O Retired
- O Other? Please specify: ____________________

Please check the academic degrees you hold and the year that degree was obtained.

	Academic degree (select all that apply)	Year degree obtained (indicate year)
Bachelor of art or science (BA/BS)	O	
Master of library science, library and information science, or information science or master's of art in library science (MLS/MLIS/MIS/AMLS)	O	
Bachelor of science in nursing (BSN)	O	
Master of science in nursing (MSN)	O	
Doctor of nursing practice (DNP)	O	
Master of social work (MSW)	O	
Medical degree (MD)	O	
Doctorate (PhD)	O	
Other degrees?<br>Please specify:	O	

SC
Supplemental content to
J Med Libr Assoc. Jul;107(3):dx.doi.org/10.5195/jmla.2019.628
www.jmla.mlanet.org
© Lauseng, Howard, Johnson 2019

During your professional degree program, did you receive any training or instruction on evidence-based medicine (EBM)/EBP?

- o Yes
- o No

If yes, indicate the type of EBP training you received. (select all that apply)

- o A full course on EBM/EBP
- o Modules within another course
- o Workshop
- o Webinar
- o Certificate program
- o Other? Please explain: ____________________

Where did you receive your professional degree?

____

Prior to participating in the EBP course, did you have previous training on EBP? (select all that apply)

- o Autodidactic (read books or articles on the topic)
- o Journal club
- o Medical Library Association continuing education (CE)
- o Other accredited CE. Please specify: ____________________
- o Training (no-CE) through presentation or workshop
- o Informal training through colleague
- o Other? Please explain: ____________________
- o No previous training

What library or information environment are you currently working in? (select one)

- o Public library
- o Academic library (other than health sciences)
- o Academic health sciences library
- o Hospital library
- o Nonlibrary academic setting
- o Nonlibrary clinical/health care setting
- o Was not employed
- o Retired
- o Other? Please specify: ____________________

Supplemental content to
J Med Libr Assoc. Jul;107(3):dx.doi.org/10.5195/jmla.2019.628
www.jmla.mlanet.org
© Lauseng, Howard, Johnson 2019

Which professional organizations do you currently belong to? (select all that apply)

- o American Library Association
- o American Medical Association
- o American Nurses Association
- o Association of College and Research Libraries
- o Medical Library Association
- o Academy of Health Information Professionals
- o Special Libraries Association
- o Other? Please list: ____________________

What topics were most valuable in the EBP course? Select the top three.

- o Evolution of Evidence-Based Practice
- o Research Study Methodology
- o Developing the Answerable Question Using Problem, Intervention, Comparison, Outcome (PICO)
- o Searching for the Evidence in the Literature
- o Introduction to Critical Appraisal
- o Use of Critically Appraised Topics (CATS)
- o Critically Appraising the Diagnostic Literature
- o Critically Appraising the Therapy Literature
- o Other, not listed above. Please list: ____________________

What activities were most valuable in the EBP course? Select the top two.

- o The unit-based lectures
- o The unit-based readings
- o Exploring websites
- o Quizzes (check your understanding)
- o Exploring the University of Illinois at Chicago (UIC) evidence-based medicine resources and guide
- o CAT exercises
- o Other, not listed above. Please specify: ____________________

SC
Supplemental content to
J Med Libr Assoc. Jul;107(3):dx.doi.org/10.5195/jmla.2019.628
www.jmla.mlanet.org
© Lauseng, Howard, Johnson 2019

Did the following assignments reinforce the unit learning objectives? Rate on a scale of 5 to 1 where 5=Strongly agree and 1=Strongly disagree, or select 0=Don't recall the assignment.

	**5= Strongly agree**	**4= Agree**	**3= Neutral**	**2= Disagree**	**1= Strongly disagree**	**0= Don't recall the assignment**
PICO assignment<br>(Objective: formulate a question using the PICO format)	O	O	O	O	O	O
Find Evidence in PubMed<br>(Objectives: conduct an effective literature search to find the best evidence, employ advanced search strategies for filtering out the best evidence in PubMed)	O	O	O	O	O	O
Critically appraise two diagnostic articles, and create a CAT for each<br>(Objectives: apply appropriate criteria in appraising an article for validity and evidence, evaluate the quality of statistical methodology in the clinical literature, and produce a formal EBM analysis using the CAT)	O	O	O	O	O	O
Critically appraise one therapy article and create a CAT<br>(Objectives: apply appropriate criteria in appraising an article for validity and evidence, evaluate the quality of statistical methodology in the clinical literature, and produce a formal EBM analysis using the CAT)	O	O	O	O	O	O
Capstone<br>(only for those completing a final summary project)	O	O	O	O	O	O

What was the primary take-away for you in participating in this EBP online course?

____

SC
Supplemental content to
J Med Libr Assoc. Jul;107(3):dx.doi.org/10.5195/jmla.2019.628
www.jmla.mlanet.org
© Lauseng, Howard, Johnson 2019

What content from the class have you been able to apply to your work responsibilities? (select all that apply)

- O Evolution of Evidence-Based Practice
- O Research Study Methodology
- O Developing the Answerable Question Using PICO
- O Searching for the Evidence in the Literature
- O Introduction to Critical Appraisal
- O Use of CATS
- O Critically Appraising the Diagnostic Literature
- O Critically Appraising the Therapy Literature
- O Other, not listed above. Please list: ____________________

Please provide an example of course content applied to your work responsibilities:

____

How has your work changed as a result of increasing your knowledge of EBP through this course?

	Yes/No		If Yes, please describe:
	Yes	No	(please describe)
New work responsibilities	O	O	
New role within the institution	O	O	
Change in job	O	O	
Other?	O	O	

Have you taken additional formalized EBP training since participating in this course?

- O Yes
- O No

If yes, please select all that apply:

- O For-credit degree course
- O For-credit non-degree course
- O Medical Library Association CE
- O Other accredited CE

JMLA Journal of the Medical Library Association

SC
Supplemental content to
J Med Libr Assoc. Jul;107(3):dx.doi.org/10.5195/jmla.2019.628
www.jmla.mlanet.org
© Lauseng, Howard, Johnson 2019

Which of these EBP topics are of interest to you for future CE opportunities? (select all that apply)

- Conducting critical appraisals
- Teaching critical appraisal skills
- Conducting systematic reviews
- Teaching systematic review process
- Teaching evidence-based decision making
- Conducting your own evidence-based research
- Other? Please describe: ____________________
